# A review of the pharmacokinetics, pharmacodynamics, efficacy, and safety of direct oral anticoagulants in patients undergoing bariatric surgery

**DOI:** 10.1016/j.rpth.2026.106799

**Published:** 2026-06-13

**Authors:** Dino Kröll, Guido Stirnimann, Dereck Wentworth

**Affiliations:** 1 Department of Visceral Surgery and Medicine, Inselspital, Bern University Hospital, University of Bern, Bern, Switzerland; 2 Medical Affairs, Cardiovascular Innovative Medicine, Johnson & Johnson, Titusville, New Jersey, USA

**Keywords:** apixaban, bariatric surgery, factor Xa inhibitors, rivaroxaban, thromboembolism

## Abstract

The role of direct oral anticoagulants for prevention of venous thromboembolism (VTE) in bariatric surgery has been unclear. Current clinical guidelines recommend against using direct oral anticoagulants in bariatric surgery but lack the inclusion of recent prospective trials. This article reviews data on the pharmacokinetics, pharmacodynamics, efficacy, and safety of rivaroxaban or apixaban prophylaxis in this setting. Targeted literature reviews identified prospective studies of rivaroxaban (*n* = 4) or apixaban (*n* = 2) and retrospective studies (*n* = 7) on VTE prophylaxis in bariatric surgery. Single-dose 10 mg rivaroxaban produced comparable systemic exposure before and after surgery, regardless of procedure type. In a randomized clinical trial, no cases of clinically overt VTE occurred with 7-day or 28-day administration of prophylactic rivaroxaban after surgery, with low rates of relevant bleeding (1.9%). A noncontrolled, nonrandomized trial with risk-adapted dosing of rivaroxaban showed no thrombosis and low bleeding risk (0.9%). Single doses of 5 mg apixaban at 1, 6, and 12 months resulted in no clinically significant changes in pharmacokinetic and pharmacodynamic parameters. A randomized controlled trial in sleeve gastrectomy patients with twice daily prophylactic apixaban or low-molecular-weight heparin resulted in no VTE and low rates of bleeding events (2%). Retrospective studies support the effectiveness and safety of rivaroxaban or apixaban in bariatric surgery patients; however, their findings are limited by study design, inconsistent dosing information, and heterogeneity of patient populations. Available evidence suggests that rivaroxaban and apixaban may be feasible options for thromboprophylaxis after bariatric surgery, although additional prospective studies are needed to confirm efficacy and safety.

## Essentials

• Direct oral anticoagulants are well established for venous thromboembolism prophylaxis in nonbariatric patients.
• Pharmacokinetic/pharmacodynamic data show no significant differences before or after bariatric surgery for rivaroxaban/apixaban.
• Rivaroxaban demonstrated efficacy/safety in sleeve gastrectomy (SG), Roux-en-Y gastric bypass, and revisional bariatric surgery patients.
• Apixaban has been shown to be efficacious and safe in SG patients to date.

## 1 Introduction

Obesity is an increasingly prevalent condition worldwide that has been linked to various health complications. Globally, the obesity rate more than doubled between 1990 and 2022, with 16% of adults aged ≥18 years classified as living with obesity in 2022 [1].

Obesity is associated with numerous comorbidities, including type 2 diabetes, obstructive sleep apnea, malignancies, stroke, coronary artery disease, atrial fibrillation, hyperlipidemia, hypertension, and venous thromboembolism (VTE) [2,3]. VTE risk increases with body mass index (BMI), with obesity increasing VTE risk by 6-fold [2].

Bariatric surgery is indicated for patients with severe obesity who cannot lose weight by nonsurgical means. Common bariatric surgery procedures include Roux-en-Y gastric bypass (RYGB), which divides the stomach and diverts stomach contents from this smaller portion into the small intestine downstream via gastrojejunostomy; sleeve gastrectomy (SG), in which the stomach size is reduced by ∼80% without anastomosis to the intestine; biliopancreatic diversion with duodenal switch, the reduction of the stomach with intestinal rerouting to reduce nutrient absorption; and gastric banding, the implantation of an adjustable silicone band around the top part of the stomach [4,5]. In 2021, SG (58%) and RYGB (26%) were the most common bariatric procedures [6]. Based on 2022 registry data, the United States had the highest number of bariatric surgery procedures performed globally [7].

Bariatric surgery is generally safe for severe obesity [5]; however, it increases the risk of VTE in the postoperative period, including deep vein thrombosis (DVT) and pulmonary embolism (PE) due to the inherent risk of obesity and the surgical procedure [8]. Guidelines from the American Society for Metabolic and Bariatric Surgery (ASMBS) recommend a combination of mechanical prophylaxis and pharmacologic prophylaxis with low-molecular-weight heparin (LMWH) to prevent VTE in patients undergoing bariatric surgery [9]. Up to 80% of postoperative VTEs occur after hospital discharge, so high-risk patients may benefit from continued VTE prophylaxis after discharge [9].

The Scientific and Standardization Committee of the International Society on Thrombosis and Haemostasis (ISTH) recommends direct oral anticoagulants (DOACs), including rivaroxaban or apixaban, as an option for VTE treatment or prevention in patients with obesity, regardless of BMI [10]. However, limited data exist on VTE prophylaxis with DOACs after bariatric surgery, and DOACs are not currently recommended in the acute postoperative period by ISTH or ASMBS [9,10]. Some small studies and case reports suggest avoiding DOACs after bariatric surgery because of potential malabsorption leading to concerns about efficacy and safety in this population [[11], [12], [13]].

The DOACs rivaroxaban, apixaban, and edoxaban are selective direct factor [F]Xa (FXa) inhibitors that inhibit free FXa and prothrombinase activity [[14], [15], [16]]. By inhibiting FXa, these DOACs decrease thrombin generation and lead to a decrease in thrombin–antithrombin complexes. Although there have been some efforts to review the role of oral FXa inhibitors for VTE prophylaxis in the setting of bariatric surgery, knowledge gaps persist due to predominantly retrospective studies and inconsistent adverse event reporting [17].

For the present narrative review, targeted literature searches were conducted in PubMed on March 2, 2026, using the following search terms with no other restrictions: (rivaroxaban or apixaban or edoxaban or DOAC) and (bariatric or “bariatric surgery”). The search identified 57 articles, of which 34 were clinical studies or commentaries on clinical studies; manual review of the literature identified 1 additional publication for inclusion. Review articles, letters, and case reports were excluded, along with studies focused on indications other than VTE, mixed indications, or VTE treatment rather than prophylaxis. In this study, we review prospective studies on the pharmacokinetics (PK), pharmacodynamics (PD), efficacy, and safety of the use of rivaroxaban (*n* = 4) or apixaban (*n* = 2) for VTE prophylaxis in bariatric surgery and summarize the results of relevant retrospective studies (*n* = 7).

## 2 Rivaroxaban PK in Bariatric Surgery

**Figure 1:**
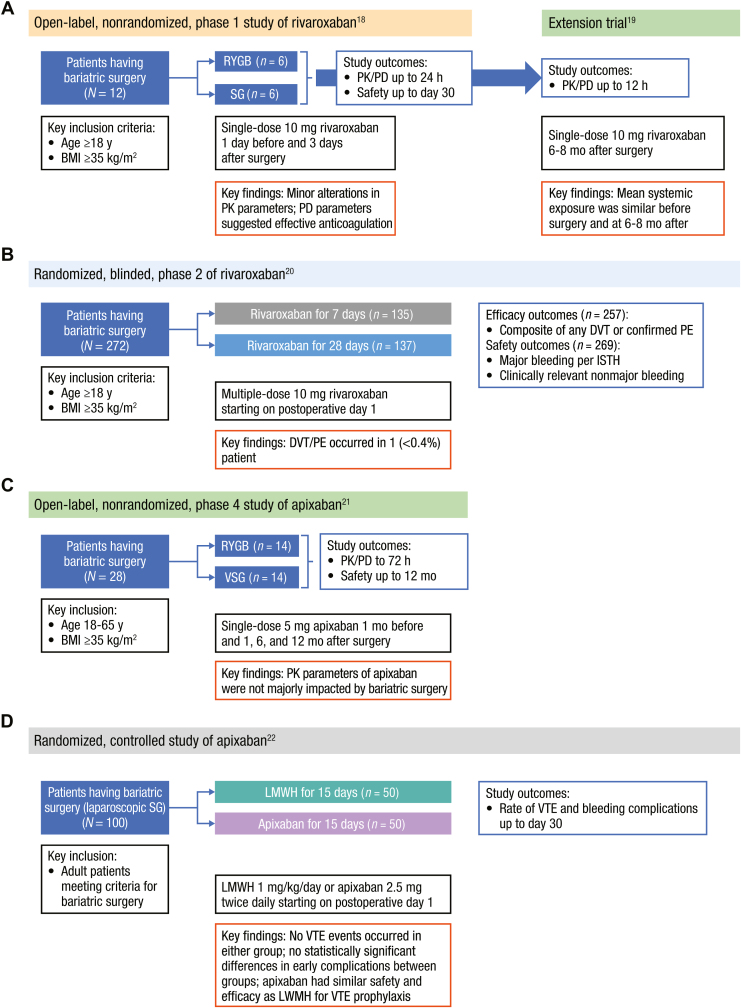
Study designs for the (A) open-label, nonrandomized, phase 1 study of rivaroxaban (short-term and extension trials), (B) randomized, blinded, phase 2 BARIVA (Bariatric Rivaroxaban) study of rivaroxaban, (C) open-label, nonrandomized, phase 4 study of apixaban, and (D) randomized, controlled study of apixaban [[18], [19], [20], [21], [22]]. BMI, body mass index; DVT, deep vein thrombosis; ISTH, International Society on Thrombosis and Haemostasis; LMWH, low-molecular-weight heparin; PD, pharmacodynamic; PE, pulmonary embolism; PK, pharmacokinetic; RYGB, Roux-en-Y gastric bypass; SG, sleeve gastrectomy; VSG, vertical sleeve gastrecomy; VTE, venous thromboembolism

Two studies assessed single-dose PK/PD parameters of prophylactic use of rivaroxaban (10 mg) in patients undergoing bariatric surgery (Figure 1A) [18,19].

### 2.1 Short-term study: Kröll 2017

The first study of rivaroxaban PK/PD in bariatric surgery patients was a phase 1, single-center, open-label, nonrandomized trial in 12 patients (RYGB, *n* = 6; SG, *n* = 6) [18]. All patients received intermittent pneumatic compression and early mobilization as standard of care. The primary endpoints were single-dose PK parameters of rivaroxaban after administration 1 day before and 3 days after RYGB or SG. Mean age was 39 years in both groups, and all patients were White; two-thirds were female. Mean BMI at baseline was higher in the SG group (44.6 kg/m^2^) than in the RYGB group (38.5 kg/m^2^).

Table 1 [18,19] presents the PK parameters before and after surgery for all patients and by type of surgery. Mean time to peak plasma concentration was delayed by 1 hour after the procedure in the RYGB group (1.5 vs 2.5 hours), but not in the SG group. Rivaroxaban half-life was decreased in the SG group from 13.1 hours before surgery to 8.9 hours after surgery. However, these differences were not considered clinically relevant [23], and no other differences in the PK profile of rivaroxaban were found after bariatric surgery compared with before surgery.

**Table 1:** Pharmacokinetic parameters of rivaroxaban before and after surgery and at midterm follow-up for all patients and subgroups by type of surgery [18,19].

Patients	Parameters	Before surgery, mean/CV	3 d after surgery, mean/CV	Ratio before vs 3 d after surgery (95% CI)	Midterm (6-8 mo) FU, mean/CV	Ratio before surgery vs midterm FU (95% CI)
Overall	AUC-μg·h/L	952.6/16.8	1095.5/16.8	0.87 (0.77-0.98)	922.4/43.2	1.0 (0.8-1.2)
	C_max_-μg/L	135.9/19.3	137.3/33.3	0.99 (0.79-1.24)	116.8/47.1	1.2 (0.9-1.5)
	t_1/2_^a^-h	12.8/35.9	11.6/58.7	1.16 (0.82-1.64)	13.5/68.8	0.9 (0.6-1.5)
	V_Z/f_^a^-L/kg	46.5/20.6	44.4/26.1	1.08 (0.99-1.18)	66.2/36.4	0.7 (0.6-0.8)^c^
	T_max_^b^-h	1.5 (0.9-4.0)	2.0 (1.0-4.0)	NA	3.0 (0.5-5.9)	NA
RYGB	AUC-μg·h/L	933.7/22.3	1029.4/7.4	0.91 (0.75-1.09)	869.1/44.5	1.1 (0.8-1.4)
	C_max_-μg/L	136.5/10.7	110.8/31.8	1.23 (0.91-1.66)	99.1/31.3	1.4 (1.0-1.9)
	t_1/2_^a^-h	12.4/42.1	15.0/60.0	0.92 (0.57-1.48)	12.9/84.5	1.0 (0.6-1.6)
	V_Z/f_^a^-L/kg	53.2/22.9	52.7/20.8	1.05 (0.91-1.21)	70.3/34.4	0.8 (0.6-0.9)^c^
	T_max_^b^-h	1.5 (0.9-4.0)	2.5 (1.0-4.0)	NA	3.0 (1.0-5.9)	NA
SG	AUC-μg·h/L	971.9/10.6	1165.8/21.9	0.83 (0.68-1.02)	978.9/45.3	1.0 (0.7-1.4)
	C_max_-μg/L	135.3/26.7	170.0/15.9	0.80 (0.59-1.08)	137.7/57.1	1.0 (0.6-1.6)
	t_1/2_-h	13.1/34.1	8.9/44.6	1.47 (0.82-2.64)	14.0/63.8	0.9 (0.4-2.4)
	V_Z/f_-L/kg	41.5/9.5	37.4/18.1	1.11 (0.95-1.29)	63.0/40.7	0.7 (0.4-1.0)^c^
	T_max_^b^-h	1.5 (1.0-4.0)	1.5 (1.0-4.0)	NA	2.0 (0.5-3.0)	NA

AUC, area under the plasma–concentration time curve from time 0 to infinity; C_max_, peak plasma concentration; CV, coefficient of variation; FU, follow-up; NA, not applicable; RYGB, Roux-en-Y gastric bypass; SG, sleeve gastrectomy; t_1/2_, terminal half-life; T_max_, time to peak plasma concentration; V_Z/f_, apparent volume of distribution during the terminal phase divided by total body weight (in kg).

a One patient was excluded from the t_1/2_ and V_Z/f_ calculations due to missing data; before surgery values from Kröll et al. 2018 [19] are reported.

b T_max_ is presented as median and range.

c Statistically significant difference (*P* <.05).

PD parameters suggested effective anticoagulation with rivaroxaban. Thrombin–antithrombin complexes decreased within 1 to 3 hours of preoperative rivaroxaban administration. The decrease was significant within 1 hour, with median levels declining about 4-fold in both surgery groups. Thrombin–antithrombin values remained low after surgery. Median levels of prothrombin fragments 1 and 2 decreased by about 2- to 3-fold in both groups within 12 hours after rivaroxaban administration.

Rivaroxaban was well tolerated in bariatric surgery patients, and all patients continued into the extension study. The only serious adverse event was a jejunal obstruction after RYGB requiring surgical revision, which was considered unrelated to rivaroxaban.

### 2.2 Extension study: Kröll 2018

In the extension trial, the primary endpoints were single-dose PK parameters of rivaroxaban after oral dosing at 6 to 8 months postsurgery [19]. The median decrease in BMI after bariatric surgery was 9.9 kg/m^2^ in the RYGB group and 12.5 kg/m^2^ in the SG group, corresponding to 76.6% and 71.4% excess weight loss and 24.2% and 29.0% total weight loss, respectively.

Mean systemic exposure (area under the plasma–concentration time curve from time 0 to infinity [AUC]) was similar before surgery and at 6 to 8 months after surgery in both groups (Table 1). The apparent volume of distribution increased significantly in both surgery groups, consistent with the reduced body weights observed. Prothrombin fragments 1 and 2 decreased after rivaroxaban administration and reached a nadir at 12 hours, with similar pre- and postoperative changes. No adverse events occurred.

## 3 Efficacy and Safety of Rivaroxaban in Bariatric Surgery

### 3.1 BARIVA trial: Kröll 2023

The BARIVA (Bariatric Rivaroxaban) study was a phase 2, randomized, assessor-blinded, multicenter trial to assess the efficacy and safety of prophylactic rivaroxaban (10 mg) after bariatric surgery in short-term (7-day; *n* = 134) vs extended (28-day; *n* = 135) prophylaxis arms (Figure 1B) [20]. Postoperative care included thromboprophylaxis with LMWH 6 to 8 hours after surgery (stopped at randomization to rivaroxaban), early mobilization, and intermittent pneumatic compression. The primary efficacy outcome was the composite of any DVT or objectively confirmed PE. The primary safety outcome was the incidence of ISTH-defined major bleeding (bleeding leading to transfusion or a decrease in hemoglobin level ≥2 g/dL during the intervention and observation periods). Clinically relevant nonmajor bleeding (CRNMB) based on the ISTH definition was defined as any bleeding requiring medical intervention, hospitalization, or face-to-face evaluation [24].

Mean age was 40 years in both groups, and most (97%) patients were White; 80% were female. Mean BMI was 41.7 kg/m^2^ in the short-term prophylaxis group and 42.6 kg/m^2^ in the extended prophylaxis group. Patients underwent RYGB (51.3%), SG (42.4%), or revisional surgery (6.3%).

The primary efficacy endpoint occurred in only 1 of 257 (0.39%) patients who underwent SG and was randomized to the extended prophylaxis group; the patient had an asymptomatic DVT. There were no cases of clinically overt DVT or PE.

Major bleeding occurred infrequently, with 1 case in the short-term prophylaxis group (0.7%) after SG and 1 in the extended prophylaxis group (0.7%) after RYGB (Table 2 [20]). CRNMB also occurred infrequently: 1 case in the short-term prophylaxis group (0.7%) and 2 in the extended prophylaxis group (1.5%); all were RYGB patients. Postoperative complications occurred in 5.4% of patients in the short-term prophylaxis group and 13.3% in the extended prophylaxis group; most postoperative complications involved surgical site infections. Type of surgery was not a factor in DVT or PE incidence, bleeding rates, or the incidence of adverse events in both prophylaxis groups.

**Table 2:** Primary and secondary safety endpoints in the BARIVA (Bariatric Rivaroxaban) study [20].

Safety endpoint	Overall study group *N* = 269		Rivaroxaban 7 d *N* = 134		Rivaroxaban 28 d *N* = 135	
Overall	*n*	% (95% CI)	*n*	% (95% CI)	*n*	% (95% CI)
Primary endpoint (major bleeding)	2	0.7 (0.2-2.7)	1	0.7 (0-4.1)	1	0.7 (0-4.1)
Secondary endpoint (CRNMB)	3	1.1 (0.4-3.2)	1	0.7 (0-4.1)	2	1.5 (0.4-5.2)
Major and CRNMB	5	1.9 (0.8-4.3)	2	1.5 (0.4-5.3)	3	2.2 (0.8-6.3)

**Roux-en-Y gastric bypass**		***N* = 138**		***N* = 69**		***N* = 69**
Primary endpoint (major bleeding)	1	0.7 (0-4.0)	0	0 (0-5.3)	1	1.4 (0.1-7.8)
Secondary endpoint (CRNMB)	3	2.2 (0.7-6.2)	1	1.4 (0.1-7.8)	2	2.9 (0.8-10.0)
Major and CRNMB	4	2.9 (1.1-7.2)	1	1.4 (0.1-7.8)	3	4.3 (1.5-12.0)

**Sleeve gastrectomy**		***N*****= 114**		***N* = 55**		***N* = 59**
Primary endpoint (major bleeding)	1	0.9 (0.0-4.8)	1	1.8 (0.1-9.6)	0	0 (0-6.1)
Secondary endpoint (CRNMB)	0	0 (0-3.3)	0	0 (0-6.5)	0	0 (0-6.1)
Major and CRNMB	1	0.9 (0.1-4.8)	1	1.8 (0.1-9.6)	0	0 (0-6.1)

**Revisional surgery**		***N* = 17**		***N* = 10**		***N* = 7**
Primary endpoint (major bleeding)	0	0 (0-18.4)	0	0 (0-27.8)	0	0 (0-35.4)
Secondary endpoint (CRNMB)	0	0 (0-18.4)	0	0 (0-27.8)	0	0 (0-35.4)
Major and CRNMB	0	0 (0-18.4)	0	0 (0-27.8)	0	0 (0-35.4)

CRNMB, clinically relevant nonmajor bleeding.

### 3.2 Single-center study: Tyselskyi 2023

Tyselskyi et al. [25] performed a prospective, single-site cohort study to assess the safety and effectiveness of rivaroxaban for VTE prophylaxis using ultrasound examination of the portal vein and veins of the lower extremities.

The incidences of VTE and adverse events were studied in 110 patients who underwent bariatric surgery and received rivaroxaban 2.5 mg twice daily to 10 mg daily for 30 days based on Caprini score starting on postoperative day 4. Average BMI was 55 kg/m^2^, and most patients underwent SG resection (84%) with mobilization 3 hours after surgery. Average follow-up was 6 months, and no clinical or radiological evidence of thromboembolic complications was observed. One patient developed a subcutaneous hematoma while receiving rivaroxaban that did not require intervention.

## 4 Pharmacokinetics, Efficacy, and Safety of Apixaban in Bariatric Surgery

### **4.1** **The open-label, nonrandomized apixabanstudy: Steele 2022**

Patients with BMI ≥35 kg/m^2^ undergoing vertical SG or RYGB received a single dose of apixaban 5 mg preoperatively and at 1, 6, and 12 months postoperatively (Figure 1C) [21]. Blood samples were collected before dosing and at 0.5, 1, 2, 3, 4, 6, 9, 12, 18, 24, 48, and 72 hours after dosing for measurement of maximum serum concentration (C_max_), time to C_max_ (T_max_), AUC from 0 to 72 hours, elimination half-life (t_1/2_), and change in FX activity. All study visits were completed by 28 patients (14 undergoing SG and 14 undergoing RYGB), with 89% being female and having a mean age of 43.6 years and mean BMI of 48.6 kg/m^2^.

AUC was higher at 1 month postoperatively compared with preoperatively, returned to values similar to preoperative values at 6 months, and decreased significantly at 12 months postoperatively (which may reflect reduced body weight), with similar patterns for both types of surgical procedures (Table 3 [21]). C_max_ and T_max_ did not change significantly between preoperative and postoperative assessments. In both surgical groups, t_1/2_ increased at 1 month, with a significant change in the RYGB group, and the reason for this effect is not clear. The t_1/2_ progressively decreased thereafter and was not significantly different at 6 and 12 months in both groups. A progressive decrease in FX activity was observed from before dosing to 3 hours after dosing and through 1, 6, and 12 months postoperatively. The decline in FX activity was more pronounced in patients undergoing SG than for RYGB. The authors concluded that bariatric surgery did not affect the PK parameters of apixaban, particularly C_max_, in a clinically significant way, but efficacy was not assessed.

**Table 3:** Pharmacokinetic parameters of apixaban preoperatively and 1, 6, and 12 months postoperatively by type of surgery [21].

Patients	Parameters	Preoperatively, mean (SD)	1 mo postoperatively, mean (SD)	6 mo postoperatively, mean (SD)	12 mo postoperatively, mean (SD)
Overall	AUC_0-72_, ng/mL/h	1009.1 (227.1)	1232.9 (434.4)^b^	1000.9 (389.5)	841.8 (297.2)^b^
	C_max_, ng/mL	83.1 (23.3)	88.3 (28.4)	83.9 (31.7)	82.4 (26.7)
	t_1/2_, h	9.50 (2.04)	11.31 (2.68)^b^	10.31 (3.14)	9.04 (2.46)
	T_max_^a^, h	2.5 (2-3)	3 (2-3.5)	2 (2-3)	2 (2-3)^b^
RYGB	AUC_0-72_, ng/mL/h	1006.9 (212.4)	1302.3 (497.0)^b^	989.0 (410.0)	840.6 (299.2)^b^
	C_max_, ng/mL	80.5 (23.5)	84.7 (35.2)	79.0 (30.2)	73.8 (22.3)
	t_1/2_, h	9.43 (1.94)	12.18 (2.90)^b^	10.53 (3.18)	9.24 (2.60)
	T_max_^a^, h	3 (2-3)	3 (3-4)	2.5 (2-3)	2.5 (2-3)
VSG	AUC_0-72_, ng/mL/h	1011.3 (249.1)	1163.5 (366.8)	1012.8 (383.0)	843.0 (306.5)^b^
	C_max_, ng/mL	85.7 (23.6)	91.9 (20.0)	88.9 (33.5)	91.0 (28.7)
	t_1/2_, h	9.57 (2.21)	10.44 (2.21)	10.10 (3.22)	8.83 (2.39)
	T_max_^a^, h	2 (2-3)	2 (1-2)^b^	2 (1-3)^b^	2 (1-2)^b^

AUC_0-72_, area under the plasma–concentration time curve from time 0 to 72 hours; C_max_, peak plasma concentration; RYGB, Roux-en-Y gastric bypass; t_1/2_, terminal half-life; T_max_, time to peak plasma concentration; VSG, vertical sleeve gastrectomy.

a T_max_ is presented as median and range.

b Statistically significant difference vs before surgery (*P* <.05).

### 4.2 Randomized controlled trial: Abdelsalam 2025

The safety and efficacy of apixaban was directly compared with LMWH in 100 adults undergoing laparoscopic SG who were randomized to receive postoperative doses of LMWH (*n* = 50; 1 mg/kg/d for 15 days, up to 120 mg/d) or apixaban (*n* = 50; 2.5 mg twice daily for 15 days; Figure 1D) [22]. The mean age was 36 years (range, 18-59), and both groups were predominantly female. The mean baseline BMI was 49 kg/m^2^ in the LMWH group and 47 kg/m^2^ in the apixaban group. Obesity-associated medical complications were prevalent in 24% of patients receiving LMWH and 36% of patients receiving apixaban.

No VTE events occurred in either group, and postoperative bleeding was encountered in 1 patient per group (2% each). Follow-up venous Doppler studies were unremarkable in both groups. Early postoperative adverse events were reported in 4% (*n* = 2/50) of the LWMH group and 10% (*n* = 5/50) of the apixaban group and included postoperative bleeding (*n* = 1/50 per group), minor leaks (*n* = 1/50, LMWH group only), and fever (*n* = 4/50, apixaban group only). Differences in early complications were not statistically significant between groups, and no mortality was reported in either group. The authors concluded that apixaban was comparable to LWMH for the prevention of VTE after laparoscopic SG with similar efficacy and safety.

### 4.3 Retrospective reports of DOAC use in bariatric surgery

Patients with a history of bariatric surgery were excluded from phase 3 studies of DOACs, and these studies did not report outcomes in patients with severe obesity [3]; however, data from nonrandomized retrospective studies are available. Table 4 [[26], [27], [28], [29], [30], [31], [32]] provides an overview of retrospective analyses of DOAC use for VTE prophylaxis in patients undergoing bariatric surgery. Given the low reported incidence of VTE-related events, these studies support the efficacy of rivaroxaban and apixaban for use in patients after bariatric surgery; low rates of thrombosis and bleeding further support the safety of their use. Importantly, these studies are limited by a lack of information on DOAC dosing and controlling for patients’ baseline characteristics. Nevertheless, these results suggest that guidance against using DOACs after bariatric surgery unnecessarily prevents patients from accessing oral FXa inhibitors as prophylactic options that provide ease of use with fewer drug interactions, fixed dosing, less monitoring, and low bleeding risk.

**Table 4:** Retrospective studies evaluating use of direct oral anticoagulants for VTE prophylaxis in patients undergoing bariatric surgery.

Study	*N*	Type of study	Methods of thromboprophylaxis	Age, y, mean (SD)	BMI, kg/m^2^, mean (SD)	Female, *n* (%)	SG, *n* (%)	RYGB, *n* (%)	Other procedures, *n* (%)	Efficacy outcomes	Safety outcomes
Rodríguez, 2020 [26]	198	Single-center, single-surgeon analysis	LMWH (enoxaparin 40 mg once daily) during hospital stay	36.3 (11)	36.2 (2.67)	135 (68)	198 (100)	0	0	PMVT: 4/198 patients (all within 30 d of surgery)	No bleeding episodes reported
	223	Single-center, single-surgeon analysis	Rivaroxaban 10 mg daily for 10 d after discharge	34.5 (10)	35.7 (3.39)	141 (63)	223 (100)	0	0	PMVT: 0/223 patients	No bleeding episodes reported
Bayat, 2022 [27]	500	Single-center analysis	Enoxaparin 2.5 mg twice daily	43.8 (3.5)	47.2 (1.7)	295 (59)	500 (100)	0	0	No VTE events reported during ≥3 mo of follow-up	Not evaluated
	500		Rivaroxaban 10 mg once daily	45.2 (3.1)	49.6 (5.6)	315 (63)	500 (100)	0	0	VTE: 1/500 (0.2%) patients was diagnosed 23 d after discharge. No reported VTE-related mortality at ≥3 mo of follow-up	
Swartz, 2023 [28]	144	Single-center, single-surgeon analysis	Rivaroxaban 10 mg daily for 30 d after discharge	42.7 (11.7)	48.0 (8.0)	80.5^a^	144 (100)	0	0	PMVT: 4/142 patients who did not receive postdischarge thromboprophylaxis vs 0/144 patients receiving rivaroxaban	Bleeding events: 4/144 patients receiving rivaroxaban; 2 patients had hematomas that did not require intervention and 2 patients returned to surgery for a staple line bleed and a rectus sheath hematoma/bleed
Guzman-Pruneda, 2024 [29]	1443	Single-center analysis	Apixaban 2.5 mg twice daily for 30 d after discharge	41 (13)	44 (7)	1101 (76)	795 (83)	148 (16)	10 (1)	No patients who received apixaban developed VTE vs 5 patients who did not receive apixaban	• Postoperative hematocrit drops of ≥6 points with clinical signs of bleeding were similar between groups (22.0% vs 22.1%) as were drops of ≥9 points (2.6% vs 2.5%) • Postdischarge bleeding events: 3 (0.3%) patients in the no apixaban group and 7 (0.5%) patients in the apixaban group
Surve, 2022 [30]	5017	3-center study	Apixaban 2.5 mg twice daily for 30 d after discharge	43.2 (12.1)	44.6 (8.5)	—	59.7^a^	4.4^a^	35.6^a^	• Thromboembolic events: 10 (0.1%) patients (5 PVT and 5 PE) • No DVTs were reported	Side effects: 90 (1.7%) patients; 44.4% menorrhagia and 42.1% rash
Galvao Goncalves, 2025 [31]	2737	Single-center case study	Apixaban 2.5 mg twice daily for 14 d after discharge	43.1 (11.5)	44.7 (6.7)	77.3^a^	2737 (100)	0	0	VTE: 3/2737 (0.1%) patients; all portal vein thrombosis	Bleeding complications: 11/2737 (0.4%) patients; most were abdominal and related to surgery
Berends, 2025 [32]	97	Retrospective cohort study	Apixaban 5 mg twice daily	57 (51-61)^b^	43.2 (39.5-46.8)^b^	62 (63.9)	22 (22.7)	69 (71.1)	6 (6.2)	No thromboembolic events occurred	No major bleeding events occurred; 1 clinically relevant nonmajor bleeding was observed

BMI, body mass index; DVT, deep vein thrombosis; IQR, interquartile range; LMWH, low-molecular-weight heparin; PE, pulmonary embolism; PMVT, portal and mesenteric venous thrombosis; PVT, portal vein thrombosis; RYGB, Roux-en-Y gastric bypass; SG, sleeve gastrectomy; VTE, venous thromboembolism.

a Percentage only reported.

b Median (IQR) reported.

## 5 Discussion

### 5.1 PK/PD and safety findings

Single doses of 10 mg rivaroxaban resulted in similar systemic exposure before and immediately after bariatric surgery, regardless of procedure type, and the PD results support clinical utility of rivaroxaban [18]. This is further supported by a recent meta-analysis, which failed to find a statistically significant difference in the overall mean change in peak plasma concentration of rivaroxaban before and after bariatric surgery [33]. Systemic exposure after a single dose at 6 to 8 months after bariatric surgery was similar to that immediately before surgery [19]. Despite excess weight loss of >71% at 6 to 8 months after surgery and an accompanying increase in volume of distribution, the degree of adipose tissue loss had no correlation with rivaroxaban systemic exposure [19]. There were also no safety concerns [18,19]. Of note, these PK/PD and safety data are based on small sample sizes [18,19]. However, the studies included multiple sampling points for PK measurements, in contrast to other PK studies of rivaroxaban that have relied on sampling only pre- and postdose (at predicted time to peak plasma concentration [T_max_]) [11], as well as the evaluation of PK using systemic exposure measures (eg, AUC) and point exposure values (eg, peak plasma concentration [C_max_]) [18,19]. Use of multiple sampling points results in more reliable determinations of all relevant PK parameters (C_max_, T_max_, and AUC) as a precondition to evaluate the anticoagulant effects of rivaroxaban due to its relatively short half-life [14,34]. AUC is the most reliable measure of drug bioavailability, whereas time to C_max_ can vary among patients from approximately 1 to 4 hours [14]. Thus, without frequent sampling near the expected peak, a PK study may underestimate the peak concentration, leading to inaccurate assumptions about absorption and anticoagulation effectiveness. Similarly, in patients undergoing bariatric surgery, single doses of 5 mg apixaban at 1, 6, and 12 months after surgery showed clinically insignificant changes in PK/PD parameters of apixaban that were not expected to affect effectiveness [21]. In fact, apixaban plasma concentrations had somewhat increased relative to baseline in the early postoperative period (1 month), potentially due to slower upper gastrointestinal motility after surgery [21]. Moreover, FX activity was found to consistently decline after administration of 5 mg apixaban at each postoperative time point measured [21]. Altogether, these results suggest that bariatric surgery may not meaningfully impact the PK/PD parameters of oral FXa inhibitors. Adequately powered, prospective efficacy trials are needed prior to routine use of rivaroxaban and apixaban in patients undergoing bariatric surgery [18,21].

### 5.2 Efficacy and safety findings

In the BARIVA study, 10 mg rivaroxaban resulted in only 1 case of the composite endpoint of DVT and PE, which was an asymptomatic DVT, and no differences were found after 7 or 28 days [20]. Likewise, no meaningful differences in rivaroxaban efficacy or safety were found across surgery types, although few revisional surgery procedures were done [20]. Rivaroxaban prophylaxis for 7 or 28 days after bariatric surgery appears to be effective and safe, and the data suggest that a 7-day regimen may be sufficient for patients at moderate risk of VTE [20]. Additionally, considering that 61% of patients had body weight >120 kg and 55% had BMI >40 kg/m^2^, this study supports rivaroxaban prophylaxis after bariatric surgery in patients with severe obesity [20].

No bleeding events were reported in the phase 1 PK and extension studies [18,19]. In the BARIVA study, major bleeding or CRNMB was rare, occurring in 5 (1.9%) patients overall [20]. These data appear comparable to trials investigating LMWH as prophylaxis for bariatric surgery, which showed VTE in 0% to 1.5% of patients and major bleeding or CRNMB in 0% to 5.6% [[35], [36], [37]]. Similarly, there were no thrombotic events and a low risk of bleeding, affecting <1% of patients in a single-center, noncontrolled, nonrandomized trial of rivaroxaban [25].

In the recently published prospective, randomized controlled trial by Abdelsalam et al. [22], apixaban was shown to have comparable safety and efficacy with LMWH for VTE prophylaxis after SG, with no VTE events and low rates of postoperative bleeding (2%) observed in both groups. These results underscore apixaban’s potential as an alternative to LMWH in patients undergoing bariatric surgery.

Importantly, these findings appear to be consistent across drugs that act on FXa, with a recent meta-analysis estimating the rate of thrombotic complications at 0.23% (95% CI, 0.14-0.39) across 7 studies investigating thrombotic outcomes of bariatric surgery using apixaban, rivaroxaban, or combination enoxaparin/rivaroxaban prophylaxis [17]. Moreover, the prevalence of minor and major bleeding events (0.33% [95% CI, 0.11-1.03]; 1.27% [95% CI, 0.45-3.54], respectively) was low across studies, further supporting the potential effectiveness and safety of oral FXa inhibitors for thromboprophylaxis in bariatric surgery.

### 5.3 Current clinical guidelines

Rivaroxaban and apixaban are approved by the US Food and Drug Administration for treatment of DVT and PE and for postoperative prophylaxis only after knee or hip replacement surgery [38]. Rivaroxaban and other oral FXa inhibitors are not currently recommended by ISTH for treatment or prevention of VTE in the acute post-bariatric surgery setting because of concerns about decreased absorption [10]. The ASMBS stresses individualization of VTE prevention protocols and mentions rivaroxaban as a possible alternative to LMWH, pending further studies [9].

The 2016 ISTH guidelines on DOAC use in patients with obesity advised against using DOACs in individuals with severe obesity (weight >120 kg or BMI >40 kg/m^2^) [39], leading to the perception that obesity alone reduces DOAC efficacy. In 2021, updated guidelines endorsed VTE treatment using standard doses of rivaroxaban or apixaban for anticoagulation, regardless of BMI and weight [10]. This change appeared to be primarily based on evidence from clinical trials and population PK modeling showing no significant influence of body weight on rivaroxaban PK, efficacy, or safety [[40], [41], [42]], as fewer supportive data were available at the time for apixaban [10].

The studies highlighted herein provide supportive evidence for rivaroxaban and apixaban in VTE prophylaxis after bariatric surgery, which could influence future ISTH guidance statements. First, PK/PD parameters of rivaroxaban and apixaban appeared to be unaffected by obesity or bariatric surgery [18,21]. Second, absorption and systemic exposure of rivaroxaban (at 6-8 months) or apixaban (at 6-12 months) after bariatric surgery were largely unchanged despite alterations to the gastrointestinal tract [19,21]. Third, no symptomatic VTE events occurred after 7 or 28 days of daily rivaroxaban administration, regardless of bariatric surgery type [20]. Although more data are needed, these results may support revising the ISTH recommendations.

### 5.4 Methodologic issues in studies of DOACs following bariatric surgery

The ISTH guidance statement recommending against use of DOACs in the acute post-bariatric surgery setting were based on PK/PD studies with small sample sizes [10]. Additionally, some studies reporting rivaroxaban peak blood levels below the expected therapeutic level after bariatric surgery were suboptimal [11,43]. In these studies, only a single “peak” blood level was measured to estimate T_max_, which may underestimate AUC and therapeutic efficacy given variability of T_max_ [44]. Blood levels should be collected at several time points near rivaroxaban’s T_max_, as absorption may be delayed postsurgery [18]. The study by Rottenstreich et al. [11] acknowledged that predicted T_max_ may inaccurately represent time to actual peak level after bariatric surgery and noted that a complete PK profile over time was not obtained.

Many researchers maintain that rivaroxaban should be avoided after bariatric surgery due to potential malabsorption in the stomach [13]. However, a study of healthy subjects who received a 20 mg rivaroxaban tablet that was finely crushed and administered via nasogastric tube found a double peak of absorption at ∼45 minutes and 4 to 6 hours after dosing, indicating absorption in the stomach and the small intestine (duodenum) [45]. All PK parameters, including exposure, assessed after nasogastric tube administration were similar to those observed with oral dosing [45], suggesting that intestinal absorption can contribute greatly to rivaroxaban absorption. Importantly, similar rivaroxaban PK parameters were observed in patients before and after gastric bypass after oral administration of a 10 mg rivaroxaban tablet (not crushed) and frequent blood monitoring over 24 hours postdose to capture both peaks of absorption. These findings support that anatomical changes, including bypass of the duodenum with RYGB, do not cause reduced absorption of rivaroxaban [18,19]. Crushing the rivaroxaban tablet is only necessary if it cannot be swallowed, but crushing the tablet is not necessary for absorption of the drug in the stomach and small intestine.

### 5.5 Limitations

Taken together, these studies suggest the feasibility of using oral FXa inhibitors for patients undergoing bariatric surgery. However, there are some important limitations to consider. The lack of available evidence for edoxaban limits understanding of its clinical outcomes related to bariatric surgery. Some of the studies reviewed included small sample sizes and single-center designs, which may limit generalizability across populations. Moreover, evidence is lacking for less frequently employed procedures, including biliopancreatic diversions and gastric banding. Altogether, additional data are needed on clinical outcomes, safety, and duration of therapy, including in patients with very high BMI (>50 kg/m^2^). Inclusion of oral FXa inhibitors in bariatric surgery registries, such as the Metabolic and Bariatric Surgery Accreditation and Quality Improvement Program of the American College of Surgeons, would also be a valuable method to gather such evidence.

## 6 Conclusions

Prospective studies support the use of prophylactic doses of rivaroxaban and apixaban for patients undergoing bariatric surgery based on PK/PD, efficacy, and safety data. Direct comparison of oral FXa inhibitors with LMWH is currently limited to a single trial. Rivaroxaban provides the convenience of once daily dosing, whereas apixaban requires twice daily administration. Although both rivaroxaban and apixaban have been prospectively evaluated in patients undergoing SG, efficacy data for gastric bypass patients are currently available only for rivaroxaban. Retrospective studies support the efficacy and safety of oral FXa inhibitors in bariatric surgery patients, but their value is limited due to methodological constraints. Further prospective studies are warranted to validate the current evidence, particularly in the context of updated clinical guidelines.
